# Revision Thoracic Syringo-Subarachnoid Shunt for Recurrent Syrinx With Syringobulbia: Technique and Surgical Video

**DOI:** 10.7759/cureus.28577

**Published:** 2022-08-30

**Authors:** Kiran Sankarappan, Andrew Pack, Arpan Patel, Benjamin Whiting, William Clifton

**Affiliations:** 1 Neurosurgery, Texas A&M College of Medicine, Bryan, USA; 2 Neurological Surgery, Cleveland Clinic Foundation, Cleveland, USA; 3 Neurological Surgery, Columbia University, New York, USA

**Keywords:** syringomyelia, neurosurgery, spine, spinal cord, syringobulbia, syrinx

## Abstract

Syringomyelia and syringobulbia continue to remain a diagnosis without widely accepted treatment paradigms. Furthermore, the currently available treatment options can be complicated by delayed symptom recurrence and the need for revision surgery. Revision intradural surgery is challenging, and currently, there is a paucity of literature describing safe techniques for revision syringotomy and shunt placement. In this technical report, we present a surgical video describing the technique of revision syringo-subarachnoid shunt placement in a 61-year-old female with a history of multiple intradural surgeries who presented with progressively symptomatic ascending syringobulbia.

## Introduction

Syringomyelia is a chronic progressive neurological condition that is characterized by a cystic fluid-filled cavity within the spinal cord that develops in the setting of abnormal cerebrospinal fluid (CSF) circulation. Patients with syringomyelia can present with a variety of symptoms that are often associated with the size and location of the cyst. Symptoms can include headache, numbness, pain, weakness, and spasticity. Primary syringomyelia refers to the development of a syrinx in the absence of an identifiable cause that is altering the normal flow of CSF. Secondary syringomyelia refers to the development of a syrinx in the setting of an identifiable cause such as Chiari I malformation, post-traumatic, infectious, and tumor [1]. When the location and extension of the syrinx involve the medulla, the diagnosis is referred to as syringobulbia. Syringobulbia, often associated with cranial nerve dysfunction, can develop as an independent lesion localized to the cervicomedullary junction or as a progression of syringomyelia, referred to as ascending syringobulbia [2].

While multiple treatment options exist for symptomatic syringomyelia and syringobulbia, the appropriate treatment algorithm remains unclear. When an identifiable etiology is clear (i.e., tumor, tethering, Chiari malformation), management is aimed at treating this underlying cause. For patients without an identifiable cause, but with imaging demonstrating evidence of abnormal CSF flow dynamics and arachnoid adhesions, a decompression with arachnolysis is recommended [3]. Decompression with the restoration of normal CSF flow has shown results in symptom improvement and radiographical resolution of syrinx [4]. In cases refractory to decompression or without imaging evidence of arachnoid webbing, shunting techniques have been employed, including syringo-subarachnoid, -peritoneal, and -pleural shunting with varying reports of success and revision rates [5]. There currently is minimal literature discussing the technical details of re-operation for recurrent syringomyelia. In this technical report, we present an operative video and description of recurrent syringomyelia treated with syringo-subarachnoid shunt.

## Technical report

A 61-year-old female presented with progressive numbness of her body below her jawline and increased difficulty with ambulation. Her past medical history was relevant for an episode of listeria meningitis treated in childhood. The patient was first diagnosed with a post-infectious syrinx localized to the T2-5 region and had undergone a T2-T5 laminectomy and myelotomy in 2010. She subsequently underwent ventriculoperitoneal shunt placement in 2016, followed by revision thoracic decompression with intradural exploration and lysis of adhesions in 2017. The patient presented to our facility in 2019 with continued complaints of gait imbalance and lower extremity numbness. Updated imaging revealed significant expansion of the syrinx to the cervicomedullary junction. In light of these progressive symptoms in the setting of multiple failed attempts at decompression, arachnolysis, and restoration of normal CSF flow, the decision was made to proceed with revision laminectomy, intradural exploration, and placement of syringo-subarachnoid shunt (see Video 1).

**Video 1:** Surgical video demonstrating the technique for revision myelotomy and placement of syringo-subarachnoid shunt

Informed consent was obtained. The patient was positioned prone on the Jackson table with a face-pillow for support. Somatosensory evoked potentials (SSEPs) and motor evoked potentials (MEPs) were monitored throughout the case. The prior incision was opened and the scar was dissected to expose the prior laminectomy defect. Meticulous dissection was employed to excise the epidural scar to facilitate intradural exploration. After appropriate exposure was obtained, the dura was opened in the midline using a #11 blade knife until the cerebrospinal fluid was identified. Using blunt dissection with a nerve hook, a plane was developed between the subarachnoid space and the spinal cord. The previous myelotomy site was identified. 4-0 nylon sutures were placed into the dura for retraction. Using a microdissector, the pia mater was freed from the arachnoid adhesions. The dural opening was extended and dissection of the arachnoid was continued including around the lateral margins of the spinal cord.

Once the arachnoid adhesions were removed, the previous myelotomy was identified, explored, and the cyst was entered. The myelotomy was then extended in the caudal direction to create room for shunt tubing in the cyst. A small silastic catheter was temporarily placed into the syrinx to ensure no adhesions would preclude placement of the syringo-arachnoid shunt. A T-shaped subarachnoid shunt was introduced into the myelotomy site with one end passed cranially and the other end caudally. The distal end of the tubing was placed into the subarachnoid space. This portion was secured to the spinal cord pia with a 6-0 polypropylene suture. The wound was then copiously irrigated. The dura was closed with a 4-0 nylon running suture. This was reinforced with several muscle patches, surgicel, and dural sealant glue. Valsalva maneuver confirmed satisfactory dural closure. The wound was subsequently closed in layers, including muscle, fascia, dermis, and skin. The patient tolerated the procedure well with immediate improvement in her numbness and bulbar symptoms. The patient was discharged to an inpatient rehabilitation facility on postoperative day 7 with follow-up imaging planned in 3 months.

## Discussion

For patients with recurrent syringomyelia after surgical intervention, shunting is an option. Without treatment, patients with syringomyelia have been noted to develop progressive symptoms including paralysis. In cases of untreated syringobulbia, patients may experience tetraplegia, respiratory depression, and death [6].

In 1997, Klekamp et al. published a retrospective study of 107 patients treated for syringomyelia between multiple institutions. Twenty-nine (27%) were managed conservatively due to stable neurological status. The other 78 patients that underwent surgical intervention had syringomyelia from varying etiologies including traumatic, inflammatory, infectious, and idiopathic. In patients with focal syrinxes (<2 levels), only 17% required revision at 5 years while 63% of patients with >2 levels of disease required revision during the same period [7].

The patient presented here experienced an exceedingly rare scenario of ascending syringobulbia following multiple surgical interventions targeted at decompression and arachnolysis. Although the exact pathophysiology of the CSF dynamics of idiopathic syringomyelia is unknown, it has been postulated that ascending syringobulbia develops as a result of the upward fluid impulses of the more caudal syrinx. Furthermore, cystic dilation noted in the medulla is proposed to represent either a progressive dilatation of the spinal central canal or a rostrally-directed rupture of a cervical cyst [8]. 

The most common types of shunts utilized in syringomyelia are syringo-subarachnoid, syringo-peritoneal, and syrgino-pleural shunts. All three types of shunts have been shown to improve symptoms in patients with syringomyelia. A recent meta-analysis by Rothrock et al. reported that at least 60% of patients will have neurological improvement following shunting. However, up to 13% of patients will undergo clinical deterioration immediately following shunting. Rates of revision ranged from 10-28% among the different shunt types while the rate of complications ranged from 23-39%. Overall, shunting is associated with high rates of complications, revisions, and risk of deterioration [9]. Often utilized as a last-line treatment modality, we advise that patients should be thoroughly informed of the potential risks and complications prior to proceeding with shunting.

## Conclusions

Syringomyelia and syringobulbia continue to remain challenging conditions to manage and treat. Surgical treatment is often targeted at the presumed underlying cause for the syrinx such as tumor, Chiari malformation, or arachnoid adhesions. In cases that are refractory to surgical decompression or idiopathic syringomyelia without an identifiable cause, syrinx shunting is a viable and effective option for treatment, although associated with a high risk and revision profile. Despite the prevalence of this condition treated by neurosurgeons, there is a paucity of operative videos detailing the microsurgical technique for revision myelotomy and syringo-subarachnoid shunt placement. This article and the included operative video can serve as a guide for surgeons presented with similar cases of recurrent syringomyelia.
